# Mechanisms of c-Myc Degradation by Nickel Compounds and Hypoxia

**DOI:** 10.1371/journal.pone.0008531

**Published:** 2009-12-31

**Authors:** Qin Li, Thomas Kluz, Hong Sun, Max Costa

**Affiliations:** Department of Environmental Medicine, New York University School of Medicine, Tuxedo, New York, United States of America; Roswell Park Cancer Institute, United States of America

## Abstract

Nickel (Ni) compounds have been found to cause cancer in humans and animal models and to transform cells in culture. At least part of this effect is mediated by stabilization of hypoxia inducible factor (HIF1a) and activating its downstream signaling. Recent studies reported that hypoxia signaling might either antagonize or enhance c-myc activity depending on cell context. We investigated the effect of nickel on c-myc levels, and demonstrated that nickel, hypoxia, and other hypoxia mimetics degraded c-myc protein in a number of cancer cells (A549, MCF-7, MDA-453, and BT-474). The degradation of the c-Myc protein was mediated by the 26S proteosome. Interestingly, knockdown of both HIF-1α and HIF-2α attenuated c-Myc degradation induced by Nickel and hypoxia, suggesting the functional HIF-1α and HIF-2α was required for c-myc degradation. Further studies revealed two potential pathways mediated nickel and hypoxia induced c-myc degradation. Phosphorylation of c-myc at T58 was significantly increased in cells exposed to nickel or hypoxia, leading to increased ubiquitination through Fbw7 ubiquitin ligase. In addition, nickel and hypoxia exposure decreased *USP28*, a c-myc de-ubiquitinating enzyme, contributing to a higher steady state level of c-myc ubiquitination and promoting c-myc degradation. Furthermore, the reduction of *USP28* protein by hypoxia signaling is due to both protein degradation and transcriptional repression. Nickel and hypoxia exposure significantly increased the levels of dimethylated H3 lysine 9 at the *USP28* promoter and repressed its expression. Our study demonstrated that Nickel and hypoxia exposure increased c-myc T58 phosphorylation and decreased *USP28* protein levels in cancer cells, which both lead to enhanced c-myc ubiquitination and proteasomal degradation.

## Introduction

Nickel (Ni) compounds have been found to cause cancer in humans and animal models and to transform cells in culture [1], [2], [3], [4]. Recent studies showed that HIF-1α antagonizes c-Myc function and inhibits VHL-deficient renal cell carcinoma (RCC) growth [5], while HIF-2α enhances c-Myc activity in WT-8 and 786-O cells that predominantly express HIF-2α (but little HIF-1α) and promotes VHL-deficient RCC tumorigenesis [6]. Given the apparent opposite effects of HIF-1α and HIF-2α on c-Myc, it is reasonable to ask (A) whether this phenomenon takes place in other cancer cells and (B) what is the mechanism? HIF-1α and HIF-2α each dimerize with constitutively-expressed HIF-1β to form transcription factor HIF-1 and HIF-2, which in turn regulates the expression of the HIF-dependent genes by binding to hypoxia-responsive element (HRE). This process is important for the survival of hypoxic cancer cells. Nickel compounds have been shown to mimic hypoxia and activate hypoxia signaling in cells by stabilizing HIF-1α through the inhibition of Prolyl hydroxylase that targets it for degradation [7].

c-Myc belongs to the Myc family of transcription factors. By modifying the expression of its target genes, c-Myc regulates numerous biological effects, such as cell proliferation, senescence, angiogenesis, metabolism, and genetic stability [8].

c-Myc mRNA and protein are generally expressed at low levels in normal proliferating cells [9], but frequently by unknown mechanisms are overexpressed in cancer cells [10]. The half life of c-Myc is very short in quiescent cells due to proteasomal degradation [11]; however, upon serum stimulation and cell cycle entry, c-Myc becomes transiently stabilized by the Ras pathway, allowing it to accumulate to high levels [12], [13]. This stabilization is not dependent on cell cycle progression [12], [13]. The entire regulatory cycle of c-Myc, from signal transduction events leading to gene expression, to stabilization, and ultimately, degradation, includes: 1) a growth stimulatory signal leading to new c-Myc protein synthesis and Ras activation; 2) Ras promotes c-Myc protein stabilization through extracellular receptor kinase (ERK)-mediated phosphorylation of serine 62 (S62); 3) Ras activation also prevents subsequent phosphorylation of threonine 58 (T58) by PI3K/Akt-mediated inhibition of glycogen synthase kinase (GSK3β), further stabilizing c-Myc and increasing c-Myc protein levels; 4) G1 phase re-activation of GSK3β allows phosphorylation of c-Myc on T58 (P-T58-Myc); 5) the dual phosphorylated form of c-Myc is recognized by the Pin1 prolyl isomerase, that catalyzes the isomerization of the Ser62-Pro63 bond; 6) protein phosphatase 2A (PP2A) can then dephosphorylate S62 [14], leading to poly-ubiquitinylation by ubiquitin ligases F box proteins (Skp2 or Fbw7) [15]; and, 7) degradation of c-Myc by the 26S proteosome.

Here we demonstrate that in cancer cell lines c-Myc was degraded during hypoxia and specifically in A549 cells this degradation was dependent upon both HIF-1α and HIF-2α. Further mechanistic studies showed that c-Myc proteasomal degradation that was initiated by hypoxia signaling was mediated by Fbw7 ubiquitin ligase in a T58-phosphorylation-dependent manner. Hypoxia signaling decreased the amount of *USP28* deubiquitinating enzyme that was bound to c-Myc by attenuating its gene expression and protein stability. These events were coincident with an increased GSK3β-independent phosphorylated T58-Myc, resulting in increased ubiquitination and degradation. In addition, Ni ions and hypoxia increased the levels of the gene silencing mark dimethylated H3 lysine 9 at *USP28* promoter region, which suppressed *USP28* gene expression, further depleting the cell of this c-Myc salvaging enzyme.

## Materials and Methods

### Cell Lines and Culture Condition

Human lung carcinoma A549 cells were cultured in Ham's F-12 K medium (Invitrogen, Frederick, MD). Human breast cancer MCF-7 cells, human colon carcinoma HCT116 wild-type cells, and HCT116 Fbw7^−/−^ cells [16] were cultured in Dulbecco's Modified Eagle Medium (DMEM, Invitrogen). Human breast cancer MDA453 and BT474 cells were cultured in DMEM/F12 medium. All media were supplemented with 10% fetal bovine serum (FBS, ATLAS Biological, Fort Collins, CO), and 1% penicillin/streptomycin (Grand Island, NY). Cells were maintained at 37°C as a monolayer in a humidified atmosphere containing 5% CO_2_. When cell density reached approximately 70–80% confluence, they were treated with the Ni ions or cultured under hypoxic conditions.

### Plasmids, Antibodies, and Other Reagents

HA-tagged full-length human USP28 expression vector cmv-HA-USP28 was kindly provided by Dr. Stephen J. Elledge [17]. pFlag-Fbw7α (human) plasmid was from Dr. Bruce E. Clurman [18]. pRC-cmv-Myc wild type plasmid and Myc-T58A mutant plasmid were from Dr. Rosalie Sears [14]. The following antibodies were used: c**-**Myc (N-262, C-33, 9E10) (Santa Cruz), P-T58-c-Myc (Upstate Cell Signaling), GSK3β (Upstate), dimethylated H3 lysine 9 (H3K9m2), Rabbit IgG, mouse IgG (Abcam), USP28 and HA (Bethyl), Flag and α-tubulin antibody (Sigma), Ubiquitin (Invitrogen), HIF-1α antibody (BD Biosciences) and HIF-2α antibody (Novus Biologicals). Primary antibody-bound proteins were detected by using appropriate horseradish peroxidase (HRP)-conjugated secondary antibody (Santa Cruz Biotechnology) and an enhanced chemiluminescent (ECL for HRP) Western blotting system (Amersham, Piscataway, NJ). MG-132 and dimethyloxalylglycine (DMOG) were purchased from Biomol International (Plymouth Meeting, PA). All other reagents were obtained from Sigma unless otherwise specified.

### Western Blot

Cells were washed with ice-cold 1×PBS twice and lysed with ice-cold radioimmuno-precipitation assay (RIPA) buffer (50 mM Tris-HCl, PH 7.4, 1% NP-40, 0.25%Na-deoxycholate, 150 mM NaCl, 1 mM EDTA) supplemented with a protease inhibitor mixture (Roche Applied Sciences, Indianapolis, IN) for 10 min on ice. The cells were then transferred to an Eppendorf tube and kept on ice for another 30 min, followed by centrifugation at 10000×*g* for 10 min. The supernatant (whole cell lysate) was collected and the concentration of protein was measured using the Bio-Rad DC protein assay (Bio-Rad, Hercules, CA). Proteins were separated over a SDS-PAGE gel, transferred to a PVDF membrane and probed with a specific antibody against the target protein. The target protein was then detected using HRP-conjugated secondary antibodies and an ECL Western blotting system.

### Quantitative Real Time-Polymerase Chain Reaction (qRT-PCR)

RNA was isolated from cells with the Trizol Reagent (Invitrogen) according to the manufacturer. The isolated RNA was purified using RNeasy Plus Micro Kit (Qiagen). RNA concentration was then determined by measuring the absorbance at 260 nm and 280 nm using NanoDrop ND-1000 (Thermo Scientific). 1 µg of RNA was then reversed transcribed to cDNA with the Superscript III First Strand Synthesis Super-Mix Kit for qRT-PCR (Invitrogen, Carlsbad, CA). Quantitative real time-PCR was performed to amplify *USP28* and *β-actin* using the following primer pairs: *USP28*: 5′- GGACCCTTCCTTTCTCCATGA -3′ (forward) 5′- AGGCTGACTGCCTGAGTAATGTC -3′ (reverse); *β-actin*: 5′- ATCGTCCACCGCAAATGCTTCTA -3′ (forward), 5′- AGCCATGCCAATCTCATCTTGTT -3′ (reverse). 2 µl (for USP28) or 1 µl (for β-actin) was used in a final volume of 20 µl PCR reaction mix containing both the forward and reverse primers (50 nM each primer), and 10 µl Syber Green Master Mix (Applied Biosystems). For each sample, PCR was performed in triplicate. PCR analyses were carried out using the following cycle parameters: 95°C for 10 min, followed by 40 cycles of 95°C for 15 sec, and 60°C for 1 min. Fluorescence was quantified, and analyses of amplification plots were performed with the 7300 Real Time PCR System (Applied Biosystems). Results were expressed using the comparative cycle threshold (C_t_) method as described by the manufacturer. The ΔC_t_ values were calculated in every sample as C_t_ (USP28) minus C_t_ (β-actin). The calculation of the relative changes in the expression level of USP28 (ΔΔC_t_) was performed by subtraction of the ΔC_t_ from the control group (normoxia: as a calibrator) to the treatment group ΔCt (hypoxia). The relative fold change was determined as follows: 2^-ΔΔCt^ with ΔΔC_t_, and the control levels were set to 1.0.

Semi-quantitative PCR was performed to amplify *Fbw7*, *Skp2* and *β-actin* using the following primer pairs: *Skp2*: 5′-ACAGTGAGAACATCCCCCAG-3′ (forward), 5′-GGTCCATAAATGATCGTGGG-3′ (reverse). *Fbw7*: 5′-GTGATAGAGCCCCAGTTCCA (forward), 5′-CCTCAGCCAAAATTCTCCAG (reverse). *β-actin*: 5′-TCACCCACACTGTGCCC ATCTACGA-3′ (forward), 5′-CAGCGGAACCGCTCATTGCCAATGG-3′ (reverse). 0.5 or 1 µl was used in a final volume of 50 µl PCR reaction mix containing both the forward and reverse primers, dNTPs (10 mM, 1 µl each), 10× reaction buffer (5 µl), and 2U of Taq DNA polymerase (Roche, Indianapolis, IN). The conditions for PCR are: 95°C for 2 min, followed by 28 cycles (for *Skp2*), or 38 cycles (for *Fbw7*) or 20 cycles (for *β-actin*) of 30 sec at 95°C, 30 sec at 60°C, and 30 sec at 70°C. An aliquot of the PCR product was analyzed over a 1% agarose gel containing ethilium bromide (EB).

### siRNA Transfection

ON-TARGET plus SMART pool siRNAs targeting human HIF-1α (NM_001530), HIF-2α (NM_001430), Fbw7 (NM_001013415, NM_018315, NM_033632), GSK3β (NM_002093) and Skp2 (NM_032637) were all purchased from Dharmacon (siRNAs targeting each gene was a pool of four independent siRNAs). Noncoding pool siRNA was also purchased from Dharmacon but the sequence was not provided (Catalog#: D-001210-03). A549 cells were seeded in a 6-well plate (2.5×10^5^ cells/well), and on the subsequent day cells were transfected with 100 pmol siRNA and 5 ul RNAi Max reagent (Invitrogen) for 48 hr, and then were treated with 1.0 mM NiSO_4_ or hypoxia (1% O_2_) for another 24 hr.

### Immunoprecipitations (IP)

Whole cell lysate was isolated as described above and the proteins of interest were immunoprecipitated from an equivalent amount of protein for each sample using the specific antibodies. Antibody was incubated on a rotator at 4°C for 2 hr, and then was immunoprecipitated with protein A/G plus agarose beads (Santa Cruz) overnight at 4°C followed by washing 4 times (5 min each time) with RIPA buffer containing protease and phosphatase inhibitors. After the last wash, the supernatant was removed and the pellet was resuspended in 40 µl of 1× Laemmli sample buffer. After boiling at 90–100°C for 5 min, samples were centrifuged to pellet the agarose beads, and the resultant supernatant subjected to Western blot analysis.

### Transient Transfection

One of these plasmids (HA-cmv-USP28, pRC-cmv-Myc wild type, Myc-T58A mutant vector, or empty vector), or all three plasmids (pRC-cmv-Myc, pFlag-Fbw7α and HA-cmv-USP28) were transiently transfected into A549 cells in a 6-well plate (for Western blot) or in a 100 mm-dish (for IP) with lipofectamine LTX reagent (Invitrogen). 24 hr after transfection, cells may be treated with 1.0 mM NiSO_4_ or hypoxia (1% O_2_) for another 24 hr. Cells were then collected and lysed with RIPA buffer and whole cell lysate was used for Western Blot analysis or immunoprecipitations.

### Chromation Immunoprecipitation (ChIP) Assay

8×10^6^ A549 cells were seeded into a 15-cm dish. On the second day, cells were treated with 1 mM NiSO_4_ or cultured in 1% O_2_ for 24 hr. After treatment, formaldehyde was added to each dish to a final concentration of 1%. After 10 min of incubation in a fume hood, 1/20 volume of 2.5 M glycine was added into each dish and incubated at room temperature (RT) for 5 min with gentle mixing. The medium was then aspirated and the cells were washed with ice-cold PBS twice. After the last wash, the cells were replenished with PBS supplemented with proteinase inhibitor and the cells were collected using a disposable scraper. The cells were spun down at 1,000×g for 5 min, and the pellet was washed with ice-cold PBS twice and lysis buffer three times. The chromatin immunoprecipitation (ChIP) assay was performed according to the Affymetrix Chromatin Immunoprecipitation Assay Protocol (Qiagen, Valencia, CA). PCR amplification of the *USP28* gene was performed using the primers as follows: 5′-CATGCCCAGGTTAAGAGCAT-3′ (forward), 5′-TTCCCATCAGGCTTTGTAGC-3′ (reverse). The conditions of PCR amplification were 95°C for 2 min and 29 cycles of 95°C for 30 sec and 56°C for 30 sec, and 72°C for 30 sec. The PCR products were then separated over a 1% agarose gel containing EB.

### Statistical Analysis

Each experiment was performed at least three times and representative data are shown. Data are reported as mean values±standard error of the mean (SEM). Statistical differences were calculated by using the two-tailed Student's t-test with error probabilities of p<0.05 to be significant.

## Results

### Ni Compounds Decreased c-Myc Protein Levels in Various Cancerous Cell Lines

**Figure 1:**
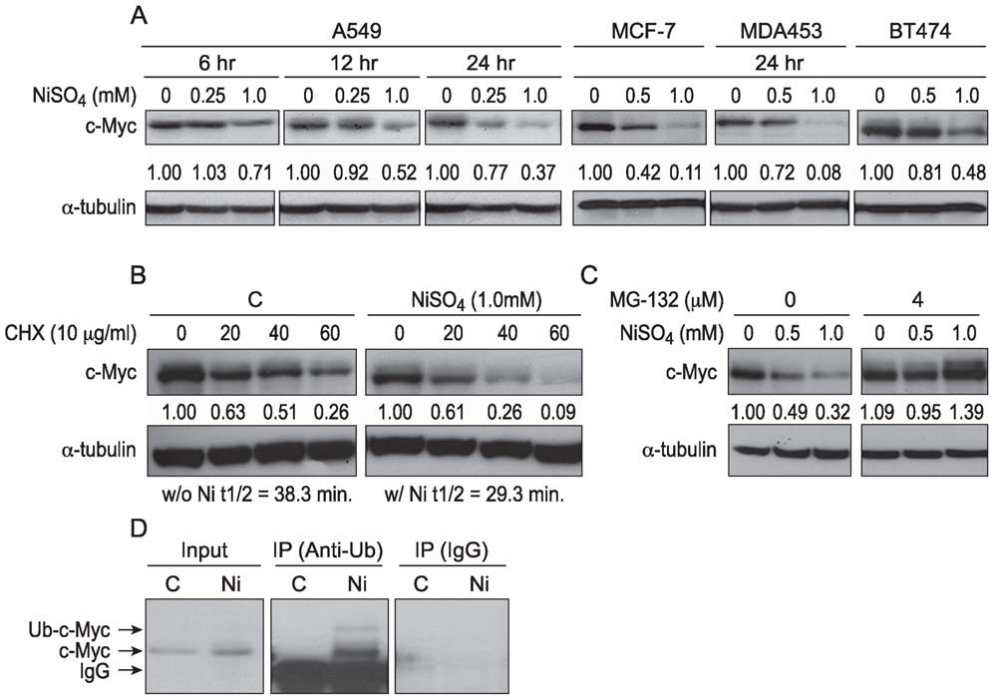
The decrease in c-Myc protein levels in A549 cells was primarily due to proteasomal degradation. (A) Various cancerous cell lines were treated with NiSO_4_ at indicated concentrations for 6, 12 or 24 hr. After treatment, the cells were lysed and whole cell lysates were analyzed by Western blot using anti-c-Myc antibody. The same membranes were then stripped and re-probed with α-tubulin antibody to assess protein loading. The densitometric data, normalized to α-tubulin level, is expressed as fold-change from control; these values are presented between the blots. (B) A549 cells were treated with 1.0 mM NiSO_4_ for 24 hr and then cycloheximide (CHX) was added to a final concentration of 10 µg/ml. Cells were lysed at selected time intervals, whole cell lysate was then isolated for Western blot. The relative intensity of the bands was quantitated using Image J software and normalized to the loading control. The numbers quantitating the intensity of the bands were given below the corresponding bands and were used to fit the exponential lines by Excel, and half-lives were calculated and shown below the figure. (C) A549 cells were pretreated with 4 µM of MG-132 for 2 hr and then NiSO_4_ was added into the medium for another 24 hr. After treatment, cells were lysed and 50 µg of whole cell lysate was analyzed by Western blotting. (D) Ni compounds increased ubiquitinated c-Myc (Ub-c-Myc) in A549 cells. A549 cells were transfected with c-Myc plasmid. 24 hr after transfection, cells were treated with NiSO_4_ (1 mM) in the presence of MG-132 (4 µM) for another 24 hr. Cells were then lysed and immunoprecipitated with anti-Ubiquitin (anti-Ub) antibody or non-specific IgG. Precipitates were analyzed by immunoblotting with anti-c-Myc (c-33) antibody (mouse).

We have studied the effects of Ni compounds on c-Myc protein levels in human lung carcinoma A549 cells. As shown in Figure 1A, c-Myc protein level was decreased by NiSO_4_ in A549 cells in a dose- and time-dependent manner. Additional cancer lines were used to investigate whether the Ni-induced c-Myc dysregulation was a general phenomenon. The results showed that Ni compounds also decreased c-Myc in other human cancer cell lines including MCF-7, DA453, and BT474 (Figure 1A).

### c-Myc Was Degraded by Ni Compounds through a Proteasome Pathway

Since c-Myc protein level decreased when a variety of cancer cells were exposed to NiSO_4,_ while the c-Myc mRNA level was not significantly changed in Ni-exposed A549 cells (data not shown), it is likely that Ni ions act to decrease c-Myc protein stability. In order to assess the change in the stability of c-Myc protein brought about by Ni ions, cells were treated with 1.0 mM NiSO_4_ for 24 hr, and then incubated with 10 ug/ml of the protein synthesis inhibitor cycloheximide (CHX). c-Myc half life was about 38.3 min in untreated cells, but it decreased by Ni treatment to 29.3 min (Figure 1B). Ubiquitin-mediated proteolysis by the 26S proteasome has been shown to be responsible for the relatively short life of the c-Myc protein [19]. To determine whether a degradation process was associated with loss of c-Myc, A549 cells were pretreated with the proteasomal inhibitor (MG-132), prior to being exposed to Ni ions. The proteasomal inhibitor prevented the nickel-induced loss of c-Myc (Figure 1C). Furthermore, as shown in Figure 1D, Ni ions increased ubiquitinated c-Myc (Ub-c-Myc) levels in A549 cells. These results indicated that Ni ions reduced c-Myc protein by activating a proteasome-dependent degradation process that shortened the life of c-Myc in the cell.

### The Degradation of c-Myc by Ni Ions Was Dependent upon HIF-1α and HIF-2α

**Figure 2:**
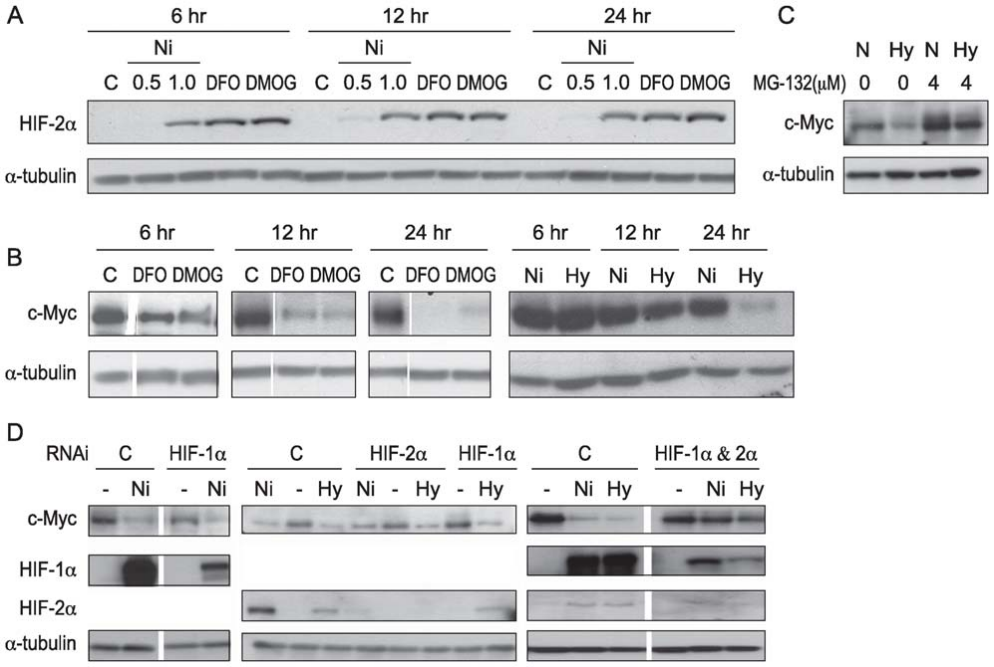
Knocking down both HIF-1α and HIF-2α attenuated c-Myc degradation by Ni ions and hypoxia in A549 cells. (A) NiSO_4_ (0.5 and 1 mM), desferrioxamine (DFO: 100 µM), and dimethyloxallyl glycine (DMOG: 1 mM) induced HIF-2α in A549 cells in a time-dependent manner. (B) 100 µM DFO, 1 mM DMOG, and hypoxia (1% O_2_) decreased c-Myc protein levels in A549 cells in a time-dependent manner. N is normoxia, while Hy is hypoxia. (C) Proteasome inhibitor MG-132 prevented c-Myc degradation by hypoxia in A549 cells. (D) A549 cells were transfected with non-coding control siRNA or siRNA targeting human *HIF-1α*, *HIF-2α* or mixture (*HIF-1α* RNAi and *HIF-2α* RNAi together) for 48 hr, and then were treated with 1.0 mM NiSO_4_ or hypoxia for another 24 hr. After treatment, cells were lysed and whole cell lysate was analyzed by Western blot. The same membranes were stripped and re-probed with α-tubulin antibody as loading control. Similar data were obtained in at least two other independent experiments; one representative blot is shown here.

Previous studies have clearly shown that Ni compounds mimicked hypoxia, by stabilizing HIF-1α in all cell line that has been studied thus far. A new finding shown in Figure 2A is that Ni ion exposure also stabilized HIF-2α in A549 cells (Figure 2A). To further understand the role of hypoxia signaling in Ni ions-induced c-Myc degradation, desferrioxamine (an Fe chelator, DFO), dimethyloxallyl glycine (an oxoglutarate analogue, DMOG), and hypoxia were utilized in A549 cells to stabilize HIF-1 and HIF-2. The results showed DFO, DMOG and hypoxia all caused c-Myc degradation in A549 in a time-dependent manner (Figure 2B). Treatment with MG-132 indicated that c-Myc was degraded through a proteasome pathway when hypoxia signaling was active in A549 cells (Figure 2C).

To study whether HIF-1 and HIF-2 were involved in Ni-induced c-Myc degradation, HIF-1α, HIF-2α or both were knocked down with RNAi in A549 cells. Knocking down HIF-1α or HIF-2α alone did not suppress c-Myc degradation that was initiated by nickel or hypoxia; however, when both were knocked down, c-Myc degradation initiated by Ni ions and hypoxia was attenuated (Figure 2D), suggesting that both were required to degrade c-Myc.

### Fbw7, but Not Skp2, Mediated c-Myc Degradation by Ni Ions or Hypoxia in A549 Cells

**Figure 3:**
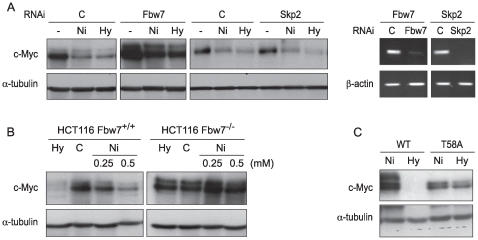
c-Myc degradation induced by Ni ions or hypoxia was mediated by Fbw7 in a T58-phosphorylation-dependent manner in A549 cells. (A) A549 cells were transfected with non-coding control siRNA or siRNA targeting human *Fbw7* or *Skp2.* At 48 hr after transfection, the cells were treated with NiSO_4_ (1.0 mM) or cultured in 1% O_2_ (Hy) for another 24 hr. Before treatment, RNA was isolated for RT-PCR (Figure 4A: right panel). (B) HCT116 and HCT116 Fbw7*^−/−^* cells were treated with NiSO_4_ (1.0 mM) or cultured in 1% O_2_ (Hy) for 24 hr. After treatment, cells were lysed and 50 µg of whole cell lysate was analyzed by Western blotting. (C) A549 cells were transfected with either wild-type (WT) or T58A-mutant c-Myc plasmids. At 24 hr after transfection, cells were cultured in 1% O_2_ (Hy) for 24 hr. Cells were then lysed and 25 µg of whole cell lysate was analyzed by Western blotting.

Fbw7 and Skp2 are ubiquitin ligases F box proteins that were found to participate in c-Myc ubiquitination and proteasomal degradation [18], [20]. To investigate whether these ubiquitin ligases were involved in hypoxia initiated c-Myc degradation, RNAi targeting human *Fbw7* or *Skp2* was transfected into A549 cells and c-Myc protein was measured after cells were treated with Ni ions or hypoxia. As shown in Figure 3A (left panel), knocking down Fbw7, but not Skp2, attenuated Ni ion- or hypoxia-induced c-Myc degradation. The right panel of Figure 3A showed that *Fbw7* and *Skp2* gene expression was efficiently repressed by RNAi transfection. To confirm that c-Myc degradation was mediated by Fbw7 in response to hypoxia signaling, we used the human colon carcinoma HCT116 cell line and a derivative cell line, HCT116 Fbw7^−/−^, in which both alleles of the *Fbw7* gene have been disrupted by homologous recombination [16]. Exposure of the parental cell line to Ni or hypoxia led to a strong decrease in c-Myc protein levels (Figure 3B). In contrast, exposure of the Fbw7-deficient cell line to Ni or hypoxia did not decrease c-Myc levels (Figure 3B). These results suggested that Fbw7 was essential for c-Myc degradation.

### The Degradation of c-Myc in A549 Cells Initiated by Hypoxia Signaling Was Dependent on T58 Phosphorylation

To further study the mechanism by which hypoxia signaling initiated c-Myc degredation, wild-type c-Myc vector or c-Myc T58A mutant vector was transfected into A549 cells. 24 hr after transfection, cells were cultured under hypoxic conditions (1% O_2_) for an additional 24 hr. Substitution of T58 of c-Myc with alanine prevented phosphorylation at that site. As shown in Figure 3C, T58A mutation stopped the hypoxia initated c-Myc degradation, underscoring the importance of T58 phosphorylation to initiate c-Myc degradation.

### Nickel Ions and Hypoxia Increased the Phosphorylation of c-Myc at T58 in A549 Cells in a GSK3Beta Independent Manner

**Figure 4:**
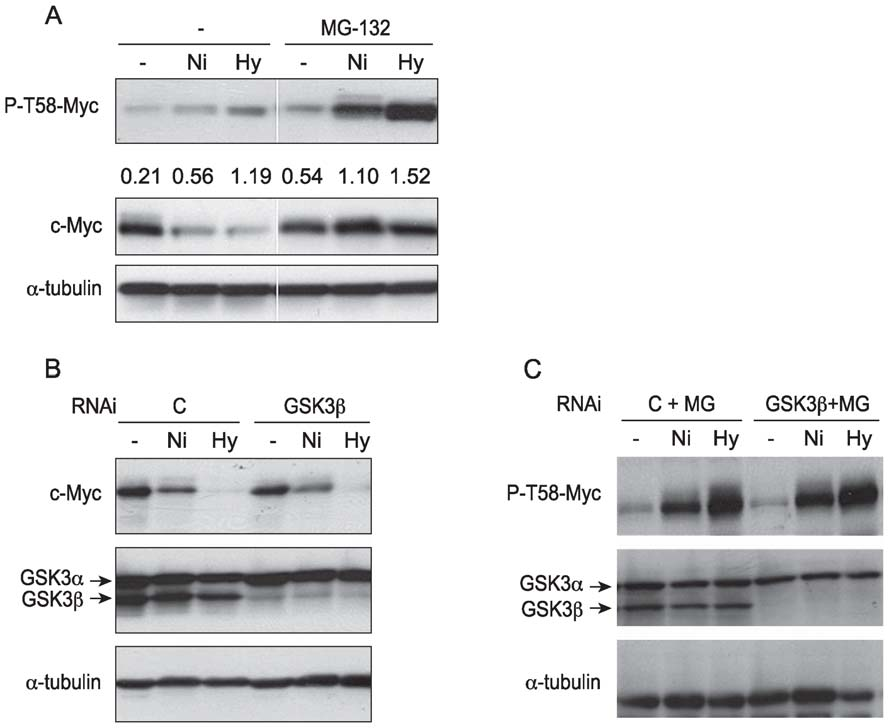
Nickel ions and hypoxia increased phosphorylation levels of c-Myc at T58 in A549 cells but not through GSK3β. (A) A549 cells were pretreated with 4 µM proteasome inhibitor MG-132 for 2 hr and then exposed to NiSO_4_ (1 mM) or cultured in 1% O_2_ (Hy) for another 24 hr. Immunoblotting of P-T58-Myc, total c-Myc, or α-tubulin was then performed as described in the Materials and Methods. The densitometric data, presented between the blots, is expressed as density-ratio of P-T58-Myc band to corresponding total c-Myc band. (B–C) A549 cells were transfected with non-coding control siRNA or siRNA targeting human *GSK3β* for 48 hr, and then were treated with NiSO_4_ (1.0 mM) or cultured under hypoxic conditions (1% O_2_) in the absence (B) or presence of 4 µM MG-132 (C) for another 24 hr. After treatment, cells were lysed and 50 µg of whole cell lysate was analyzed by Western blotting with the indicated antibodies. Similar data were obtained in at least two other independent experiments; one representative blot is shown here.

The steady state level of c-Myc ubiquitination depends on an enzymatic ubiquitin conjugation and deconjugation. To examine the mechanism by which Ni ions produce a steady sate higher amount of Ub-c-Myc, the cellular levels of phosphorylated c-Myc at T58 (P-T58-Myc) were measured in Ni ion-exposed cells. A549 cells were treated with 1 mM NiSO_4_ or hypoxia in the absence or presence of MG-132 for 24 hr. After treatment, the cells were lysed and 50 µg of protein was analyzed by Western blotting using a specific antibody to P-T58-Myc. Ni ions and hypoxia each increased P-T58-Myc levels in A549 cells, especially in the presence of MG-132 (Figure 4A). Additionally, previous studies in this laboratory demonstrated that total cellular pools of ubiquitin were not altered by Ni ions [21].

GSK3β is the only known kinase that phosphorylates c-Myc at T58 [22]. The role of GSK3β in Ni-induced c-Myc degradation was examined using siRNA targeting human *GSK3β*. Even though GSK3β protein expression was successfully repressed in A549 cells, its absence did not prevent Ni- or hypoxia-induced c-Myc degradation (Figure 4B), nor decrease the level of P-T58-Myc in the presence of MG-132 (Figure 4C). These results argue that GSK3β was not involved in c-Myc degradation in A549 cells, and that other kinases that phosphorylate c-Myc at T58 were involved in this pathway.

### Ni Ions or Hypoxia Decreased USP 28 c-Myc De-Ubiquitinating Enzyme, Contributing to a Higher Steady State Level of c-Myc Ubiquitination and Subsequent Degradation in A549 Cells

**Figure 5:**
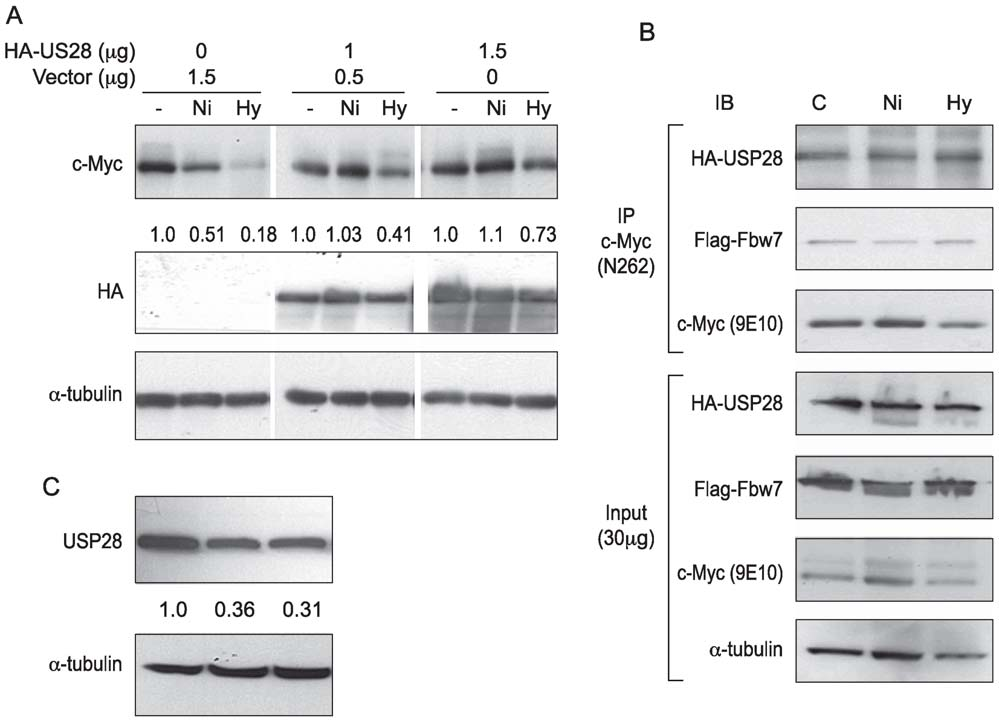
The decrease of *USP28* protein levels induced by Ni ions or hypoxia contributed to c-Myc degradation in A549 cells. (A) A549 cells were transfected with 1.0 µg or 1.5 µg HA-tagged *USP28* plasmid or empty vector. 24 hr after transfection, cells were treated with NiSO_4_ (1.0 mM) or cultured in 1% O_2_ (Hy) for another 24 hr. Cells were then lysed and protein levels were analyzed by immunoblotting. The densitometric data (of c-Myc), normalized to α-tubulin level, is expressed as fold-change from control; these values are presented between the blots. (B) Ni ions or hypoxia did not induce dissociation of USP28 or Fbw7 from c-Myc. A549 cells were co-transfected with pRC-cmv-Myc, HA-USP28, and Flag-Fbw7α-expressing plasmids. 24 hr after transfection, cells were treated with NiSO_4_ (1.0 mM) or cultured in 1% O_2_ in the presence of MG-132 (4 µM) for another 24 hr. After treatment, cells were lysed and immunoprecipitated with anti-c-Myc (N-262) antibody. Precipitates were analyzed by immunoblotting with indicated antibodies. (C) A549 cells were treated with NiSO_4_ (1.0 mM) or cultured in 1% O_2_ for 24 hr. Cells were then lysed and protein levels were analyzed by immunoblotting.

Degradation of c-Myc by Fbw7 can be reversed by the ubiquitin-specific protease, *USP28*, which can form a ternary complex with c-Myc and Fbw7α, in the nucleoplasm. To investigate whether *USP28* was involved in c-Myc degradation initiated by hypoxia, the HA-cmv-*USP28* expressing vector or corresponding empty vector was transfected into A549 cells. Following transfection, cells were treated with 1 mM NiSO_4_ or hypoxia for 24 hrs, then lysed and c-Myc protein levels were measured. As shown in Figure 5A, overexpression of *USP28* in A549 prevented the loss of c-Myc, suggesting that *USP28* was involved in c-Myc degradation.

To further explore these events, the effect of nickel ions on the de-ubiquitination process of c-Myc was examined. Nickel ions or hypoxia could impact the de-ubiquitination process by directly inhibiting *USP28* activity or indirectly decreasing *USP28* activity through either the dissociation of *USP28* from the c-Myc-Fbw7α complex or by a reduction of cellular *USP28* levels. The possible dissociation of *USP28* from the c-Myc-Fbw7α complex by Ni ions or hypoxia was investigated. A549 cells were transfected with c-Myc expression plasmids, HA-tagged *USP28*, and Flga-tagged Fbw7a and either not treated or exposed to 1 mM NiSO_4_ or hypoxia in the presence of 4 µM proteasomal inhibitor MG-132. After 24 hr of treatment, the cells were lysed and equal quantities of protein were utilized for co-IP with anti-c-Myc (N-262) antibody. Ni ions or hypoxia did not impair *USP28* association with the c-Myc-Fbw7α complex (Figure 5B). However, *USP28* protein expression was decreased by Ni ions and hypoxia in the A549 cells (Figure 5C). These outcomes indicated that a decreased *USP28* protein level caused less *USP28* binding to the c-Myc-Fbw7α complex, and sequentially increased the steady state ubiquitination levels of c-Myc.

### Hypoxia Decreased *USP28* Protein Level through Degradation and Transcriptional Repression in A549 Cells

**Figure 6:**
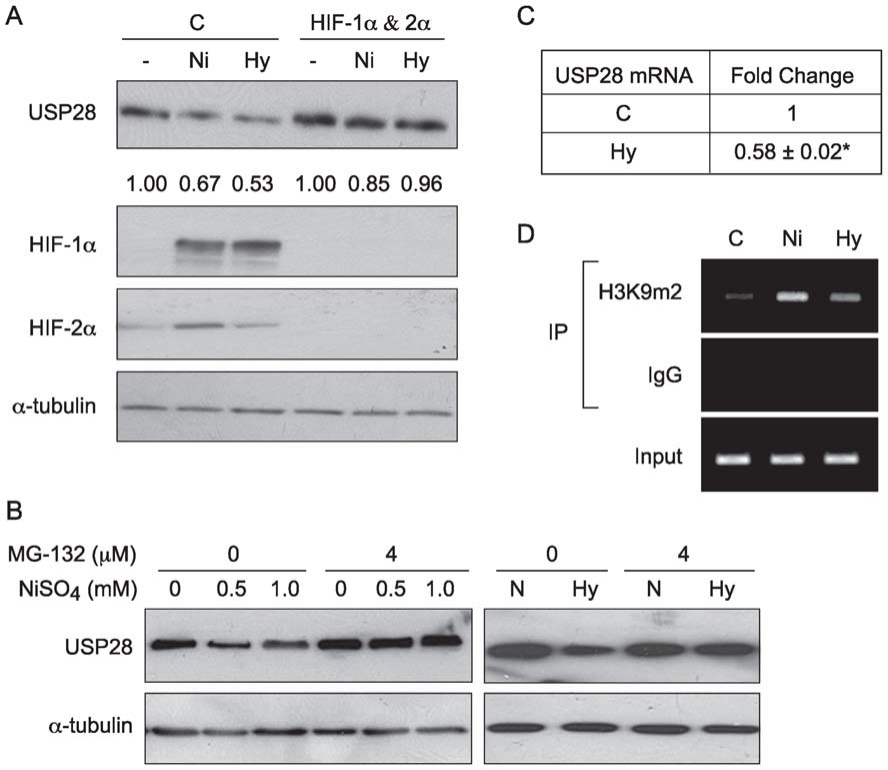
*USP28* protein level was decreased by hypoxia signaling via both protein degradation and transcriptional repression in A549 cells. (A) A549 cells were transfected with non-coding control siRNA or siRNA targeting human *HIF-1α* and *HIF-2α* (*HIF-1α* RNAi and *HIF-2α* RNAi mixture) for 48 hr, and then were treated with 1.0 mM NiSO_4_ or cultured in 1% O_2_ (Hy) for another 24 hr. (B) A549 cells were pretreated with 4 µM proteasome inhibitor MG-132 for 2 hr and then exposed to 1 mM of NiSO_4_ or cultured under 1% O_2_ for another 24 hr. After treatment, cells were lysed and 50 µg of whole cell lysate was analyzed by Western blotting. (C) Hypoxia decreased *USP28* mRNA levels in A549 cells. A549 cells were cultured in 1% O_2_ for 24 hr. After treatment, RNA was isolated for quantitative Real Time-PCR. Two tailed Student's T-test was used to analyze the fold change. Data are expressed as mean±SEM, obtained from three independent experiments; n = 3 in each experiment; *statistically significant change (p<0.05) when compared to control samples. (D) A549 cells were treated with 1 mM NiSO_4_ or cultured under hypoxic conditions (1% O_2_) for 24 hr. After treatment, the chromatin from ∼2×10^7^ cells was isolated and subjected to a ChIP assay as described in the Materials and Methods. Input DNA fractions were amplified by PCR to assess the chromatin loading.

Since both NiSO_4_ and hypoxia reduced *USP28* protein level, we asked whether HIF-1α and HIF-2α were involved in this decline. As shown in Figure 6A, knockdown of both HIF-1α and HIF-2α attenuated the decrease of USP28 protein level induced by Ni ions or hypoxia. To determine whether a degradation process was associated with decrease of *USP28* protein level, A549 cells were pretreated with a proteasomal inhibitor (MG-132), prior to their exposure to Ni ions or hypoxia. The proteasomal inhibitor prevented the loss of *USP28* protein (Figure 6B). To further study the mechanism of the hypoxia induced loss of cellular *USP28* protein, real time quantitative PCR was conducted to assess the effect of hypoxia on *USP28* gene expression. Hypoxia exposure significantly decreased *USP28* mRNA levels, (Figure 6) indicating that both degradation and gene repression were involved.

### Nickel Ions and Hypoxia Increased the Dimethylation of H3K9 in A549 Cells at the USP28 Promoter

Although little is known regarding the mechanisms by which the expression of some genes are repressed by hypoxia signaling, recent studies suggested that epigenetic mechanisms may be involved. Nickel ions and hypoxia both increased global levels of H3K9m2 [23], [24], [25], a critical mark for gene silencing. In support of these findings, another study showed that Ni ions induced *gpt* transgene silencing by increasing H3K9m2 levels at the promoter of the *gpt* gene [24]. To investigate whether the Ni-induced increase in H3K9m2 occurred at *USP28* promoter region, a ChIP assay using anti-H3K9m2 antibody was performed. Ni ions and hypoxia increased the dimethylation of H3K9m2 at the promoter of the *USP28* gene in A549 cells (Figure 6D). To investigate whether HIF-1α interacts with the *USP28* promoter, since this factor was required for the loss of *USP28*, another ChIP assay using anti-HIF-1α antibody was conducted. However, there was no observable interaction (between HIF-1α and the *USP2*8 promoter region) induced by hypoxia signaling (data not shown). HIF binding to HRE has only been reported as a mechanism for gene activation by hypoxia, the downregulation of *USP28* should not result from HIF binding to HRE. However, it should be noted that there are HRE boxes in the promoter of *USP28*. These results indicated that the increase of H3K9m2 played an important role in the repression of *USP28* gene expression induced by hypoxia.

## Discussion

Because of its critical role in cell growth, cellular c-Myc levels must be tightly controlled, but on some occasions such as in certain cancers, c-Myc has often been found to be overexpressed. However, we observed a dose-dependent decline of c-Myc protein following acute exposure to Ni compounds in a number of tumorigenic cell lines (A549, MCF-7, MD453, and BT474 cells). More detailed studies showed that the decreased c-Myc protein level was due to an increased Ub-c-Myc and sequential proteasomal degradation. It has been well known that Ni compounds mimic hypoxia and activate/stabilize HIF-1α [7]. A new finding reported here is that Ni compounds, DFO, DMOG, and hypoxia all stabilized HIF-2α protein level in A549 cells. Furthermore, DFO, DMOG and hypoxia decreased c-Myc in A549 cells suggesting that hypoxia functions in the decline of c-Myc.

The role of HIF-1α and HIF-2α in c-Myc degradation induced by Ni ions or hypoxia was verified in A549 cells. It was observed that knocking down HIF-1α or HIF-2α alone failed to block the decrease in c-Myc protein levels initiated by Ni ions or to hypoxia; c-Myc protein degradation ceased only when both HIF-1α and HIF-2α were removed. Mounting experimental evidence indicates that there are distinct functions for HIF-1α and HIF-2α during tumor growth [26], [27], and that there is little redundancy between the two α subunits [28]. However, our findings suggest that HIF-1α and HIF-2α do seem to share in the degradation of c-Myc. This “shared” function status might be specific to the hypoxic state. This was confirmed by the finding that knocking down both HIF-1α and HIF-2α failed to give rise to an increase in c-Myc protein levels above background values at normal oxygen tension. This suggests that the HIFs are not involved in the regulation of constitutive c-Myc protein stability in tumorigenic cells under normoxic conditions.

In cancerous cells, c-Myc is frequently over-expressed and stabilized by an unknown mechanism for promoting cell proliferation and mitochondrial respiration through increasing mitochondrial biogenesis [29]. In contrast, HIF-1 and HIF-2 have already been shown to inhibit mitochondrial biogenesis [5]. Previous studies have suggested that the destruction of c-Myc may be linked to its activity as a transcription factor [19], [30]. Accordingly, the opposing effects of HIFs and c-Myc on mitochondrial biogenesis may require the HIFs to repress c-Myc activity to ensure efficient attenuation of mitochondrial biogenesis, one manifestation of which may be the proteasomal degradation of c-Myc (mediated by HIFs). Thus, a greater understanding as to how HIFs induce c-Myc degradation may provide useful information for developing novel strategies to target tumor hypoxia and regulation of mitochondrial activity in cancer cells.

Two pathways for ubiquitin-dependent proteolysis are critically important for regulating c-Myc turnover: Fbw7 and Skp2. The results here demonstrated that hypoxia signaling was dependent upon Fbw7, but not Skp2, ubiquitin ligase. Mutating the T58 of c-Myc with alanine prevented the loss of c-Myc, suggesting that hypoxia signaling degraded c-Myc in a T58-phosphorylation-dependent manner. Further investigation of the underlying mechanism revealed that P-T58-Myc levels were dramatically increased after exposure to Ni ions or hypoxia (in the absence or presence of MG-132), which in turn would result in increase Ub-c-Myc levels. However, GSK3β, which has been reported to be the only enzyme to catalyze the phosphorylation of c-Myc at T58, did not appear to be involved in the degradation of c-Myc induced by hypoxia. In addition, knocking down GSK3β did not decrease P-T58-Myc levels. This paradoxical result indicated that GSK3β was not the only enzyme to phosphorylate c-Myc at T58 in A549 cells.

Previous studies have shown that degradation of c-Myc by Fbw7α was antagonized by an ubiquitin-specific protease, *USP28* [31]. Because c-Myc is continuously ubiquitinated and de-ubiquitinated in the nucleoplasm, Ni ions (and hypoxia) might also be able to regulate c-Myc stability through effects upon *USP28* activity and/or association with Fbw7. An earlier study showed that the degradation of c-Myc by UV irradiation was mediated by the dissociation of *USP28* from Fbw7α and c-Myc [32], demonstrating that *USP28* was involved in the degradation of c-Myc. Although further studies did not show that hypoxia signaling impaired the association of *USP28* with Fbw7α and c-Myc, cellular *USP28* protein levels were decreased by Ni ions and hypoxia in A549 cells. This decline in total protein resulted in decreased endogenous *USP28* binding to Fbw7α and c-Myc, which increased the levels of Ub-c-Myc.

As mentioned above, Ni ions or hypoxia degraded c-Myc by activation of HIF-1α and HIF-2α. Since *USP28* was shown to be regulated by Ni ions and hypoxia, HIFs may function in this process. Knocking down both HIF-1α and HIF-2α attenuated the decrease in *USP28* levels attributable to hypoxia signaling. Further studies showed that *USP28* proteins were also degraded through a proteasomal pathway and *USP28* mRNA level was significantly decreased by hypoxia. Therefore, both protein degradation and transcriptional repression contributed to a decrease in *USP28* protein.

Previous studies have shown that both Ni ions and hypoxia induced an increase in global levels of dimethylated histone H3 lysine 9 (H3K9m2) in mammalian cells [24], [25]. H3K9 dimethylation has been shown to correlate with gene repression by either recruiting heterochromatin protein 1 (HP1) to chromatin, or leading to histone deacetylation in both H3 and H4 [33], [34]. Here, it has been demonstrated that Ni ions and hypoxia increased H3K9m2 levels at the *USP28* promoter and repressed *USP28* gene expression.

The enzyme-catalyzed methylation of histone lysine is controlled by both a methylation and a demethylation process. Hypoxia and Ni ions have been reported to increase global H3K9m2 mostly by the oxidative histone demethylation processes [24], [25]. A Newly-identified H3K9m2-specific demethylases, i.e., JmjC-containing JHDM2 proteins, are iron- and α-ketoglutarate-dependent oxygenases [35]. Although studies showed that JHDM2A (also known as JMJD1A) was up-regulated in response to low oxygen levels *in vitro* and *in vivo* [36], its enzymatic activity should be inhibited when O_2_ was as low as 1%. In addition, it was found that Ni ions inhibited JHDM2A enzyme activity *in vitro* by interfering with the binding of iron with the enzyme [37].

In summary, this study demonstrates that Ni compounds and hypoxia induced proteasomal degradation of c-Myc through HIF-1α and HIF-2α involving both Fbw7 and T58-phosphorylation. There were two scenarios that resulted in an increase in ubiquitinated c-Myc levels in A549 cells: the first involved an increased phosphorylation of c-Myc at T58; and the second involved a decrease in *USP28* protein levels partially mediated by HIF-1 and HIF-2. The reduction of *USP28* protein by hypoxia signaling is due to both transcriptional repression and protein degradation. Both Ni ions and hypoxia increased the levels of dimethylated H3 lysine 9 at the *USP28* promoter and repressed its expression.
